# Lack of Association between Adverse Pregnancy Outcomes and Zika Antibodies among Pregnant Women in Thailand between 1997 and 2015

**DOI:** 10.3390/v13081423

**Published:** 2021-07-22

**Authors:** Nicole Ngo-Giang-Huong, Charline Leroi, Dahlene Fusco, Tim R. Cressey, Nantawan Wangsaeng, Nicolas Salvadori, Natedao Kongyai, Wasna Sirirungsi, Marc Lallemant, Prasert Auewarakul, Woottichai Khamduang, Gonzague Jourdain

**Affiliations:** 1 Maladies Infectieuses et Vecteurs: Écologie, Génétique, Évolution et Contrôle (MIVEGEC), Agropolis University Montpellier, Centre National de la Recherche Scientifique (CNRS), Institut de Recherche Pour le Développement (IRD), 34394 Montpellier, France; Tim.cressey@phpt.org (T.R.C.); Nantawan.wangsaeng@phpt.org (N.W.); Nicolas.Salvadori@phpt.org (N.S.); Gonzague.Jourdain@ird.fr (G.J.); 2 Associated Medical Sciences (AMS)-PHPT Research Collaboration, Chiang Mai 50200, Thailand; charlineleroi.bx@gmail.com (C.L.); wasna.s@cmu.ac.th (W.S.); marclallemant@gmail.com (M.L.); 3 Department of Medical Technology, Faculty of Associated Medical Sciences, Chiang Mai University, 110 Inthawaroros Road, Sripoom, Muang, Chiang Mai 50200, Thailand; natedaok@gmail.com; 4 Department of Medicine, Tulane University, 1430 Tulane Avenue, New Orleans, LA 70112, USA; dfusco@tulane.edu; 5 Department of Microbiology, Faculty of Medicine Siriraj Hospital, Mahidol University, 2 Wanglang Road, Bangkoknoi, Bangkok 10700, Thailand; prasert.aue@mahidol.ac.th

**Keywords:** Zika virus, pregnant women, adverse pregnancy outcomes, neonates, Zika virus serology, dengue virus serology, Thailand

## Abstract

Data about Zika virus infection and adverse pregnancy outcomes in Southeast Asia are scarce. We conducted an unmatched case-control study of Zika virus (ZIKV) serology in pregnant women enrolled in human immunodeficiency virus (HIV) or hepatitis B virus (HBV) perinatal prevention trials between 1997 and 2015 in Thailand. Case and control groups included women with and without adverse pregnancy outcomes. Plasma samples collected during the last trimester of pregnancy were tested for ZIKV IgG/IgM and Dengue IgG/IgM (Euroimmun, AG, Germany). Case newborn plasma samples were tested for ZIKV IgM and ZIKV RNA (Viasure, Spain). The case group included women with stillbirth (*n* = 22) or whose infants had microcephaly (*n* = 4), a head circumference below the first percentile (*n* = 14), neurological disorders (*n* = 36), or had died within 10 days after birth (*n* = 11). No women in the case group were positive for ZIKV IgM, and none of their live-born neonates were positive for ZIKV IgM or ZIKV RNA. The overall ZIKV IgG prevalence was 29%, 24% in the case and 34% in the control groups (Fisher’s exact test; *p* = 0.13), while the dengue IgG seroprevalence was 90%. Neither neonatal ZIKV infections nor ZIKV-related adverse pregnancy outcomes were observed in these women with HIV and/or HBV during the 18-year study period.

## 1. Introduction

The Zika virus (ZIKV) is transmitted to humans primarily through bites of Aedes mosquitoes that are found in tropical and subtropical regions. Most people with acute ZIKV infection present no symptoms; however, ZIKV became an emerging public health problem following reports from the Pacific and South America that ZIKV infection during pregnancy was associated with congenital microcephaly and other neurologic disorders [1,2].

Indirect evidence of circulating ZIKV infection in Southeast Asia was provided in the early 1960s with a study of the neutralizing activity of sera collected from Southeast Asian residents against various arthropod-borne viruses [3]. Over the following decades, ZIKV may have spread unnoticed, and its circulation has been poorly documented. In Thailand, the first molecular evidence of ZIKV dates back to 2006 [4] and its widespread circulation to 2012 [5]. A recent phylogenetic reconstruction of the ZIKV genome sequences obtained from patients between 2016 and 2017 suggested a continuous circulation of ZIKV since 2002 [6].

Data assessing ZIKV infection and adverse pregnancy outcomes are scarce, raising concern about the threat of unnoticed Zika-related microcephaly and neurological disorders in Thailand [7]. Between 1997 and 2015, our research team in Thailand has conducted multiple human immunodeficiency virus (HIV) or hepatitis B virus (HBV) perinatal transmission prevention studies [8,9,10,11,12]. Using stored samples and clinical data collected from pregnant women and their infants over this period provided a unique opportunity to assess the relationship between adverse pregnancy outcomes and ZIKV antibodies.

## 2. Materials and Methods

### 2.1. Design and Study Population

This was an unmatched case-control study of ZIKV serology and dengue virus serology in women with/without adverse pregnancy outcomes and their infants enrolled in five clinical studies to prevent perinatal HIV or HBV transmission in Thailand between 1997 and 2015 (PHPT-1, -2, -4 and -5, and iTAP) using different combinations and durations of antiviral drugs [8,9,10,11,12].

We confined those adverse pregnancy outcomes potentially associated with primary ZIKV infection to include perinatal mortality (stillbirth, spontaneous abortion, and death within 10 days after birth), infant microcephaly (head circumference below the first percentile for gestational age and sex/clinically reported by the investigator), or neurological disorders diagnosed within the first year of life and reported as a serious adverse event. Case group included all women with any of these adverse pregnancy outcomes. Controls were randomly selected among the other women with no such adverse outcomes.

### 2.2. Zika Virus and Dengue Virus Serology Testing

The interpretation of ZIKV serology in dengue-endemic settings is challenging due to the cross reactivity between flaviviruses, in particular antibodies directed towards the virus envelope. For this reason, we analyzed the antibody responses to the nonstructural protein (NS1) of ZIKV. Since ZIKV IgM can persist several months after infection [13,14], testing for ZIKV IgM at the beginning of the third trimester of pregnancy would allow for the retrospective identification of acute maternal infection during the first or second trimester. Stored plasma samples collected during the last trimester of pregnancy were analyzed for IgM and IgG antibodies against NS1 of ZIKV using commercial kits reported to have a very high specificity (100% overall specificity of ZIKV IgG ELISA, 95% confidence interval: 95.9–100.0) (Anti-Zika virus Elisa, Euroimmun, Germany) [15]. As dengue is endemic in Thailand, we also tested dengue NS1 serology (IgG and IgM Anti-Dengue Virus Elisa, Euroimmun, Germany) to document women’s exposure to Aedes mosquitoes, the common vectors for Zika and dengue viruses. Plasma samples collected from all live newborns of the case group were tested for ZIKV IgM and RNA (Viasure multiplex real-time PCR detection, Zaragoza, Spain) to detect a neonatal infection as a result of an in utero ZIKV transmission.

### 2.3. Statistical Considerations

We hypothesized that the adverse outcomes of interest would be more frequent in women infected by ZIKV during the first trimester of pregnancy than in control women. Assuming that 40% of controls would test positive for ZIKV IgG, we calculated that comparing 80 women in the case group and 80 women in the control would provide ≥76% power to detect a ≥20% difference in the proportion of women with ZIKV IgG between the 2 groups, based on a two-sided Fisher’s exact test at a 0.05 significance level.

The proportions of women with ZIKV IgG were computed among the case and control groups along with Clopper–Pearson 95% confidence intervals (CI). Categorical variables were compared using Fisher’s exact test and continuous variables using the Wilcoxon rank-sum test. Dengue IgG and ZIKV IgG test results were compared using McNemar’s test. Univariable and multivariable exact logistic regression models were used to assess the association between ZIKV IgG and pregnancy adverse outcomes, taking into account potential confounders, i.e., demographic, geographic and temporal characteristics of women and their pregnancies (maternal age, gestational age at delivery, parity, multiple pregnancy, geographic origin, season, educational level, preterm, sex, birth weight and body length).

## 3. Results

### 3.1. Study Population Characteristics

Of 4181 HIV-infected and 319 HBV-mono-infected pregnant women, 84 HIV-infected and three HBV-mono-infected women had an adverse pregnancy outcome. The case group included women with stillbirth (*n* = 22) or whose infants had microcephaly (*n* = 4), a head circumference below the first percentile (*n* = 14), neurological disorders within the first year of life (*n* = 36), or who had died within 10 days after birth (*n* = 11). Eighty-seven control women, including 80 HIV-infected and seven HBV-mono-infected women, were randomly selected. The median age of women was 25.1 years (IQR: 22.1–29.0) in the case group and 25.3 years (IQR: 22.7–28.0) in the control group (*p* = 0.88). Thirty-three of 59 (63%) women in the case group and 66 of 73 (90%) women in the control group had a CD4 cell count below or equal to 350 cells/mm3 (*p* = 0.11) (Table 1). Twenty-six infants among the 65 (41%) in the case group and 41 among the 87 (47%) in the control group were female (*p* = 0.51). Thirty-seven of 57 live born infants (63%) in the case group and 66 of the 73 (90%) in the control group had a birth weight of ≥2500 g (*p* = 0.001) (Table 1).

### 3.2. Women and Infants Zika Testing

None of the mothers of the caseor control groups tested positive for ZIKV IgM. Twenty of 87 (23%; 95% CI, 15% to 33%) women in the case group and 30 of 87 (34%; 25% to 45%) in the control group tested positive for ZIKV IgG (*p* = 0.13) (Table 2).

**Figure 1:**
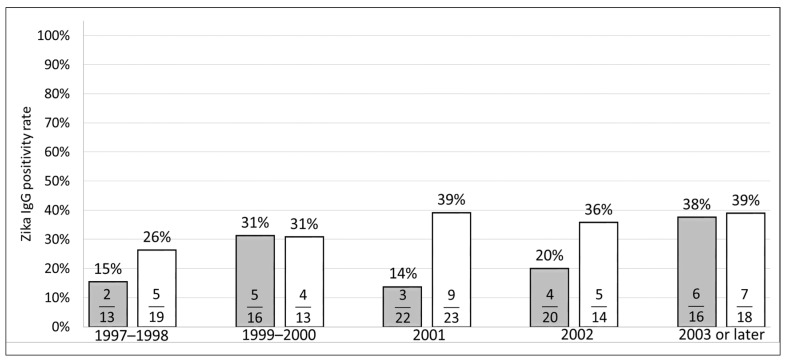
Zika virus IgG serology between 1997 and 2015.

The ZIKV IgG prevalence appeared similar across case categories. There was no evidence of a change in the ZIKV IgG positive rate between time periods in cases (*p* = 0.18) and controls (*p* = 0.26) (Figure 1).

In the univariable and multivariable logistic regression analysis, there were no significant associations between the occurrence of individual adverse outcomes and ZIKV IgG positivity after adjusting for potential confounders. There was no association between the level of the CD4 cell count and ZIKV IgG positivity; the median (IQR) CD4 cell count was 366 (270 to 526) cells/mm^3^ in ZIKV IgG positive women versus 340 (237 to 534) cells/mm^3^ in ZIKV IgG negative women (*p* = 0.59).

None of the live-born infants of cases tested positive for ZIKV IgM or RNA at birth.

### 3.3. Women Dengue Serology

None of the cases or controls tested positive for dengue IgM. Eighty of 87 (92%) cases and 76 of 87 (87%) controls tested positive for dengue IgG (*p* = 0.46) (Table 3). Of the 174 women, 49 (28%) tested positive for both dengue IgG and ZIKV IgG, 107 (61%) positive for dengue IgG only, one (1%) positive for ZIKV IgG only, and 17 (10%) negative for both (McNemar’s test, *p* < 0.001) (Table 3).

## 4. Discussion

Our study assessed the exposure to ZIKV and the relationship between adverse pregnancy outcomes and positive ZIKV IgG serology among women enrolled in HIV or HBV perinatal prevention studies between 1999 and 2015 in Thailand.

There was no association between exposure to ZIKV and adverse pregnancy outcomes. We found no evidence that the 87 case women had an acute ZIKV infection during pregnancy and transmitted the virus in utero to their offspring. Indeed, all cases tested negative for ZIKV IgM and all live infant cases tested negative for ZIKV IgM and ZIKV RNA at birth. Though ZIKV IgM testing may be less reliable in pregnant women [16], testing newborns for IgM and RNA has been used to define laboratory-confirmed microcephaly in neonates. Our finding is consistent with the low number of Zika-related microcephalic infants reported in Thailand in the last five years [17,18,19].

We found that 29% of pregnant women had been exposed to ZIKV, but we found no association between positive ZIKV IgG serology and the occurrence of adverse pregnancy outcomes. This suggests that immunity to ZIKV may have developed before conception, possibly during infancy or childhood as reported in the centralized ZIKV testing study conducted by the National Institute of Health in Thailand between 2016 and 2017 [6]. Our ZIKV IgG results are consistent with the estimated circulation of ZIKV in Thailand since 2002 [6]. Our data do not suggest a change in the prevalence of ZIKV IgG over time and may reflect a continuous exposure to ZIKV in Thailand between 1997 and 2015. Sporadic ZIKV infection cases have been reported across Thailand between 2012 and 2014 [5]; however, the reported number of ZIKV infections likely constitutes the tip of an iceberg. The number of ZIKV infections may be underestimated as most ZIKV infections are asymptomatic, symptoms could be misinterpreted as those of dengue or other viral infections, or due to the collection of blood samples long after the onset of symptoms.

Our results of dengue IgG serology prevalence were consistent with a dengue-endemic setting, and the seroprevalence of ZIKV IgG suggested that exposure to ZIKV was lesser than that to dengue viruses in Thailand, even though they share the same Aedes mosquito vectors. Individuals infected with dengue virus usually present a higher viremia (7–8 log10 copies cDNA/mL) [20] as compared to those infected with ZIKV (5 log10 cDNA copies/mL) [21], which may lead to a more effective dengue infection cycle in Aedes mosquitoes; in turn, more Aedes mosquitoes would be infected with dengue virus and thus be responsible for relatively more transmissions to humans. Another hypothesis to support the lower ZIKV IgG serology prevalence is that prior immunity to dengue virus may protect women from ZIKV infection. In vitro experiments showed that broadly neutralizing antibodies isolated from patients recovering from dengue infection could also neutralize ZIKV [22]. However, the higher rate of ZIKV IgG positivity among women positive for dengue IgG does not suggest a protective effect of dengue IgG but the common exposure to the same Aedes mosquito vector. Moreover, a recent study in Taiwan suggested that pre-existing dengue immunity facilitated ZIKV infection [23], which could result from an antibody-dependent-enhancement mechanism [24].

The association between dengue IgG positivity and ZIKV IgG positivity could be interpreted as a possible cross-reactivity of dengue IgG with ZIKV NS1 protein despite the high specificity of the assays used [15]. Studies assessing the specificity of Euroimmun NS1 dengue and the ZIKV IgG assays that we used in our study showed a high specificity among patients with primary dengue compared to secondary dengue [25,26,27]. In our study, this could not be ascertained, but, given the large geographic distribution of the dengue virus in Thailand and the high seroprevalence of dengue-specific antibodies in children [28], dengue infection may predate ZIKV infection.

Our study has some limitations. First, our study population was composed of pregnant women with HIV (most often acquired at an adult or adolescent age) or HBV (most often acquired in early infancy). However, HIV is not known to interfere with ZIKV or dengue viruses [28], and we did not find an association between CD4 cell count levels and ZIKV IgG positivity. Nevertheless, interferonopathies or other immune dysregulation/immune suppressions, caused by chronic viral infection, may protect against ZIKV-mediated teratogenicity [29]. Additionally, we did not observe a difference in ZIKV IgG prevalence among HBV-infected women. Second, we may have missed a few cases of Zika infections since IgM usually persists for a few months [14]. Additionally, only stillbirth, spontaneous abortion and infant microcephaly were considered, while the spectrum of adverse pregnancy outcomes arising from Zikavirus infections, such as ocular (chorioretinal atrophy), musculoskeletal (clubfoot) and intrauterine growth restriction, is broader in the absence of microcephaly [30]. However, it is unlikely that the few additional potential cases would have modified our results. Third, the cross-reactivity between dengue and ZIKV NS1 could not be completely ruled out; no molecular testing was performed to identify the virus since the plasma samples were collected at a time likely distant from infection, and no neutralization antibody testing was performed to ascertain the antibody response to the Zika or dengue virus. Nevertheless, the observed ZIKV IgG prevalence (24% in cases and 34% in controls) was consistent with the positivity rate of ZIKV neutralizing antibodies, 22.2% (for a 90% cutoff and a plaque reduction neutralization titer ≥20) found in normal healthy Thais [31]. The prevalence of ZIKV IgG we observed likely reflects the actual exposure of the population to ZIKV as most ZIKV infections are asymptomatic or symptoms may be misinterpreted as those of dengue infections. Further studies of the seroprevalence of Zika Ab in cohorts of young children may contribute to a better understanding of the circulation of the ZIKA virus.

## 5. Conclusions

Our study found that ZIKV immunity was common in pregnant women in Thailand. There was no evidence that the adverse pregnancy outcomes observed during 1997–2015 were related to congenital ZIKV infections. Additionally, adverse pregnancy outcomes were not associated with a maternal ZIKV antibody status.

## Figures and Tables

**Table 1:** Characteristics of mothers and neonates in Case and Control Groups.

Characteristics	Case Group (N = 87)	Control Group (N = 87)	Total (N = 174)	*p*-Value ^a^
**Mothers**				
Infection, n (%)				0.33
HIV	84 (97%)	80 (92%)	164 (94%)	
Hepatitis B virus	3 (3%)	7 (8%)	10 (6%)	
Gestational age at testing, median (IQR)	30.4 (27.41–32.1)	28.3 (26.4–31.0)	29.0 (27.0–31.8)	**0.006**
Age (years), median (IQR)	25.1 (22.1–29.0)	25.3 (22.7–28.0)	25.2 (22.4–28.3)	0.88
CD4 ≤ 350 cells/mm^3^, n (%)	33/77 (43%)	45/80 (56%)	78/157 (50%)	0.11
**Neonates**	**N = 65**	**N = 87**	**N = 152**	
Gestational age, n (%)				**0.006**
Term (≥37 weeks)	49 (75%)	80 (92%)	129 (85%)	
Sex, n (%)				0.51
Girls	27 (42%)	41 (47%)	68 (45%)	
Head circumference for GA and sex, n (%)				**<0.001**
Normal	32/64 (50%) ^b^	75 (86%)	107/151 (71%)	
Below -2 SD	32/64 (50%) ^b^	12 (14%)	44/151 (29%)	
Birth weight (g), n (%)				**<0.001**
≥2500	37/59 (63%)	66/73 (90%)	103/132 (78%)	

^a^ Fisher’s exact test for categorical variables and Wilcoxon rank-sum test for continuous variables. ^b^ Of the 18 infants with clinically-reported microcephaly or with a head circumference below the first percentile, all had a head circumference below -2 SD for gestational age and sex. Of the 36 infants with neurological disorders within the first year of life, 29 had normal head circumference and 7 had a head circumference below -2 SD. Of the 11 infants who died within ten days after birth, 3 had normal head circumference, 7 had a head circumference below -2 SD and 1 had no head circumference data reported. SD: Standard deviation, IQR: Interquartile range, GA: Gestational age. *p*-values marked with bold indicate statistically significant *p*-values.

**Table 2:** Seroprevalence of Zika and dengue IgG among pregnant women with adverse pregnancy outcomes and controls.

	Cases Number (Number Tested)	%	Controls Number (Number Tested)	%	Total Number (Number Tested)	%	*p*-Value ^a^
Zika IgG positive ^b^: All	20 ^c^ (87)	23%	30 ^d^ (87)	34%	50 (174)	29%	0.13
Stillbirth	5 (22)	23%	–	–			
Infant death after birth	2 (11)	18%	–	–			
Microcephaly or Small head circumference <1st percentile	4 (18)	22%	–	–			
Neurological disorders	9 (36)	25%	–	–			
Dengue IgG positive ^b^: All	80 (87)	92%	76 (87)	87%	156 (174)	90%	0.46
Stillbirth	19 (22)	86%	–	–			
Infant death after birth	10 (11)	91%	–	–			
Microcephaly or Small head circumference <1st percentile	16 (18)	89%	–	–			
Neurological disorders	35 (36)	97%	–	–			

^a^ Fisher’s exact test. ^b^ Defined as ≥16 Relative Units/mL. ^c^ Including 4 between 16–22 Relative Units/mL. ^d^ Including 5 between 16–22 Relative Units/mL.

**Table 3:** Association between Zika IgG and Dengue IgG.

		Dengue IgG Status		
		Positive	Negative	Total
Zika IgG status	Positive	49 (28%)	1 (1%)	50 (29%)
	Negative	107 (61%)	17 (10%)	124 (71%)
	Total	156 (90%)	18 (10%)	174 (100%)

*p*-Value from McNemar’s test < 0.001.

## Data Availability

The data presented in this study are available upon request from the corresponding authors.
